# Gene expression profile of long non-coding RNA EVF-2 in medulloblastoma cell lines and tissue samples

**DOI:** 10.1186/1753-6561-7-S2-P61

**Published:** 2013-04-04

**Authors:** Ricardo Bonfim-Silva, Thaís VCA Pimentel, Elvis T Valera, Carlos A Scrideli, Fernando S Ramalho, Hélio R Machado, Dimas T Covas, Aparecida M Fontes

**Affiliations:** 1 Department of Genetics/Faculty of Medicine of Ribeirao Preto, University of Sao Paulo, Ribeirao Preto, SP, Brazil; 2 Bloodcenter of Ribeirão Preto/ Faculty of Medicine of Ribeirao Preto, São Paulo University, Ribeirão Preto, SP, Brazil; 3 Department of Pediatrics/Faculty of Medicine of Ribeirao Preto, University of Sao Paulo, Ribeirao Preto, SP, Brazil; 4 Department of Pathology/Faculty of Medicine of Ribeirao Preto, University of Sao Paulo, Ribeirao Preto, SP, Brazil; 5 Department of Surgery and Anatomy/Faculty of Medicine of Ribeirao Preto, University of Sao Paulo, Ribeirao Preto, SP, Brazil; 6 Department of Clinical Medicine/Faculty of Medicine of Ribeirao Preto, University of Sao Paulo, Ribeirao Preto, SP, Brazil

## Background

Long non-coding RNAs (lncRNAs) are molecules that have been recently described to participate in tumor progression, however, they remain poorly characterized and their role in brain cancers, such as medulloblastoma (MDL) has not been studied in detail as yet. This study aimed to assess the gene expression profile of a lncRNA named EVF-2 in MDL tissue samples and cell lines.

## Materials and methods

We used two human MDL tissue samples, three human MDL cell lines (UW473, UW472 and DAOY), three human cerebellum tissue samples and two human cerebellum primary culture. The cell lines were characterized morphologically by light microscopy and immunophenotypically by flow cytometry. MDL cell lines and tissue samples, as well as, control cerebellum primary culture and tissue were evaluated for gene expression profile of the EVF-2 lncRNA gene by Quantitative Real Time PCR.

## Results

MDLcell lines are morphologically presented as polygonal and fibroblastoid cells. Immunophenotypically, MDL cell lines showed a high percentage (70-99%) for CD44, CD73, CD105, CD166 and CD29 and a low or absence (0-4.2%) for CD144, CD31, CD34 and CD45. MDL cell lines were different for CD271 (3,26-26,9%), CD24 (20-64%), CD146 (34-90%), CD90 (3,7-99%), CD140b (0,3-6%), and were positive for the cancer stem cell marker CD133 (2,9-5,3%). The EVF-2 lncRNA showed 81 ± 13 times more expressed in MDL tissue samples when compared to cerebellum tissue and 119 ± 48, 476 ± 74, 227 ± 33 times more expressed in MDL cell lines UW402, UW473 and DAOY respectively, when compared to cerebellum primary culture.

## Conclusion

These data demonstrate that MDL cell lines present a immunophenotype somewhat different for some markers and that EVF-2 lncRNA is more expressed in both human MDL cell lines and tumor tissue when compared to cerebellum primary culture and tissue, respectively.

## Financial support

FAPESP and Hemocentro de Ribeirão Preto-HCFMRP/USP.

